# Renal functions in pediatric patients with beta-thalassemia major: relation to chelation therapy: original prospective study

**DOI:** 10.1186/1824-7288-36-39

**Published:** 2010-05-25

**Authors:** Enas A Hamed, Nagla T ElMelegy

**Affiliations:** 1 Department of Physiology, Faculty of Medicine, Assiut University, Assiut, Egypt; 2 Department of Biochemistry, Faculty of Medicine, Assiut University, Assiut, Egypt

## Abstract

**Background:**

In β-thalassemia, profound anemia and severe hemosiderosis cause functional and physiological abnormalities in various organ systems. In recent years, there have been few published studies mainly in adult demonstrating renal involvement in β-thalassemia. This prospective study was aimed to investigate renal involvement in pediatric patients with transfusion dependant beta-thalassemia major (TD-βTM), using both conventional and early markers of glomerular and tubular dysfunctions, and to correlate findings to oxidative stress and iron chelation therapy.

**Methods:**

Sixty-nine TD-βTM patients (aged 1-16 years) and 15 healthy controls (aged 3-14 years) were enrolled in this study. Based on receiving chelation therapy (deferoxamine, DFO), patients were divided into two groups: group [I] with chelation (n = 34) and group [II] without chelation (n = 35). Levels of creatinine (Cr), calcium (Ca), inorganic phosphorus (PO_4_), uric acid (UA) and albumin were measured by spectrophotometer. Serum (S) levels of cystatin-C (S_CysC_) and total antioxidant capacity (S_TAC_) and urinary (U) levels of β_2_-microglobulin (U_β2MG_) were measured by immunosorbent assay (ELISA). Urinary N-acetyl-beta-D-glucosaminidase (U_NAG_) activity and malondialdehyde (U_MDA_) were measured by chemical methods. Estimated glomerular filtration rate (eGFR) was determined from serum creatinine.

**Results:**

In patient with and without chelation, glomerular [elevated S_CysC_, S_Cr_, U_albumin_/Cr and diminished eGFR]; and tubular dysfunctions [elevated S_UA_, S_PO4_, U_NAG_/Cr, U_β2MG_/Cr] and oxidative stress marker disturbances [diminished S_TAC _and elevated U_MDA_/Cr] were reported than controls. In patients with chelation, S_CysC _was significantly higher while, S_TAC _was significantly lower than those without chelation. In all patients, S_CysC _showed significant positive correlation with S_Cr _and negative correlation with eGFR; S_TAC _showed significant positive correlation with eGFR and negative correlation with S_Cys_C, S_Cr_, U_NAG_/Cr; U_MDA_/Cr showed significant positive correlation with U_albumin_/Cr, U_β2MG_/Cr, U_NAG_/Cr.

**Conclusions:**

Our data confirm high frequency of glomerular and tubular dysfunctions in TD-βTM pediatric patients which could be attributed to oxidative stress and DFO therapy.

## Background

β-thalassaemia major (βTM), is a type of chronic, inherited, microcytic anemia that is characterized by impaired biosynthesis of the β-globin leading to accumulation of unpaired α-globin chain. Although the prognosis for patients with thalassaemia has greatly improved in recent decades with the use of modern newborn screening, blood transfusions and iron chelation therapy; multi-organ dysfunctions is still common. Patients with thalassemia are known to have severe cardiopulmonary, reticuloendothelial, and other major systems dysfunction, but renal involvement has received little attention. Increased renal plasma flow and failure of urine concentration ability had been reported in adult subjects with β-thalassemia since 1975 [1]. Also, renal tubular acidosis had also been reported in patients with thalassemia [2]. In recent years, there have been few published studies demonstrating proteinuria, aminoaciduria, low urine osmolarity, and excess secretion of the proximal tubule damage markers, such as N-acetyl-beta-D-glucosaminidase (NAG) activity in such patients [3-5]. Shortened red blood cells (RBCs) life span, rapid iron turnover, and tissue deposition of excess iron are major factors responsible for functional and physiological abnormalities found in various forms of thalassemia. On the other hand, deferoxamine (DFO) therapy has been proved to be nephrotoxic and induce dose-dependent proximal tubular dysfunction by an unknown mechanism [6,7]. Thus, the necessity of monitoring renal damage in thalassemic patients receiving DFO therapy has been well-recognized.

Reports investigating renal dysfunction in thalassemic have been limited in number, mainly studying adult patients. Additionally, most of them have not assessed, early markers of glomerular and tubular functions, such as serum (S) cystatin-C (S_CysC_), urinary (U) β_2_-microglobulin (U_β2MG_) and U_NAG_. Early identification of patients at high risk of developing renal damage is of great importance as it may allow specific measures to be undertaken that will delay the progression of renal injury and thus reduce the incidence of renal impairment. Therefore, the aim of the present study was to: 1) investigate the presence of glomerular and/or tubular dysfunctions in children and adolescent with transfusion dependant (TD) βTM, without any overt renal diseases, using both conventional and early markers of glomerular and tubular dysfunctions. 2) To correlate the findings to oxidative stress markers and iron chelation therapy with DFO.

## Patients and Methods

Sixty-nine follow up pediatric patients with TD-βTM recruited to Hematology Clinic of Pediatric Hospital, Faculty of Medicine, Assiut University, Assiut, Egypt, were randomly selected to participate in this prospective study during the period from January 2008 to June 2009. The diagnosis of βTM was based on standard criteria [6]. The study was approved by the Ethical Committee of Faculty of Medicine, Assiut University and informed consent was obtained in every case from their legal guardians.

All patients were in a stable phase of their disease with regular erythrocyte transfusion/1-2 months (TD) since early childhood to maintain pretransfusional hemoglobin levels above 9 g/dL. Patients were divided into 2 groups, group I (n = 34) on regular iron DFO chelation therapy (20-60 mg/kg/day usually administered subcutaneously via a battery-operated portable pump over a period of 8-12 hours overnight, for 5-7 nights per week) to maintain serum ferritin level below 1000 ng/mL and group II (n = 35) without chelation therapy due to failure of treatment as a result of hypersensitivity to DFO or non-compliance. Failure of treatment was defined as an increase of serum ferritin levels >1000 ng/mL with respect to the previous values, confirmed by two determinations. Those patients who were unable to afford its prohibitive cost or unwilling to receive daily regularly rigorous subcutaneous infusions of DFO were considered as non-compliant. Demographic and clinical conditions included age, sex, weight, height, age of puberty, disease duration, number of blood transfusions/year, history of splenectomy, post-splenectomy duration, chelation therapy were obtained by interview and chart review. Patients were splenectomized when the transfusion requirements of packed red blood cells increased to 180 mL/kg/year, presence of hypersplenism signs such as leucopenia or thrombocytopenia, or presence of large spleen. Fifteen age-, sex-, body mass index (BMI)-matching healthy volunteer subjects attending the child health promotion clinic of hospital were recruited in this study as controls. Clinical examination was implemented for all participants.

Children and adolescence with clinical or laboratory evidence of renal pathology, systemic illness (cardiac, thyroid, hepatic diseases, diabetes mellitus or sepsis, etc.) or the need of renal replacement or diuretic therapy or history of intake of trimethoprim, corticosteroids or cephalosporin in the past 7 days, were excluded from this study.

Patients were instructed to fast overnight before attending the clinic in the morning and advised to abstain from taking any medications (including chelation), vitamin or mineral supplements for the previous 24 hours. Just before blood transfusion, fasting venous blood and fresh second-morning midstream urine samples were collected from all the participants (between 7-8 a.m.) for hematological and biochemical analysis. Serum and urinary samples were recovered and immediately aliquot and frozen at -70°C till use. Glomerular functions were assessed by measuring serum and urinary creatinine (Creatinine-Jaffe enzymatic assay) and urinary albumin by colorimetrically (Egypt Company for Biotechnology, Cairo, Egypt). Patients were considered to have preclinical glomerular damage if urinary albumin/creatinine ratio was 2.5-30 mg/mmol.Cr and to have glomerular proteinuria if urinary albumin/creatinine ratio was >30 mg/mmol.Cr [8]. S_CysC _levels were measured by quantikine human cystatin-C immunosorbent assay (ELISA) kit [Cat. No. DSCTC0; R&D systems, Inc., Minneapolis, MN, USA] [9]. Estimated glomerular filtration rate (eGFR) was calculated using Schwartz formula for children [10]: eGFR (ml/min/1.73 m^2^) = height (cm) × constant/serum creatinine (mg/dL), where height was expressed in "cm" and constants was 0.44 (for children < 2 years) and 0.55 (for children ≥2 years). Renal dysfunction was defined as eGFR < 90 mL/min/1.73 m^2 ^[11]. Tubular functions were assessed by measuring serum and urinary calcium (Ca), inorganic phosphorus (PO_4_) and uric acid (UA) by colorimetric method (Egypt company for biotechnology, Cairo, Egypt); β_2_MG was measured by an ELISA kit [Cat. No. ORG5BM; Orgente Dignostika, GmbH, Mainz, Germany] [12] and U_NAG _by chemical method [13]. In order to minimize urine flow rate on urinary enzyme levels, enzymes levels were expressed as ratio of enzyme activity to urinary creatinine level. Markers of oxidative stress were assessed by measuring serum total antioxidant capacity (S_TAC_) (Biodiagnostic, Giza, Egypt) by specific ELISA kit [14] and urinary malondialdehyde (U_MDA_) by chemical method [15]. The lower detection limits were 0.31 mg/dL (0.027 mmol/L) for creatinine, 0.2 ng/mL for CysC, 2.0 mg/dL for Ca, 1.0 mg/dL for PO_4_, 1.0 mg/dL (0.06 mmol/L) for UA and 0.2 μg/mL for β_2_MG.

### Data analysis

Statistical Science for Social Package (SPSS Inc, USA) software computer program version 12 was used for data analysis. Data were presented as mean ± standard deviation (SD) or number and percentage (n, %) as appropriate. For comparison of two groups the nonparametric test for independent variables was used while, comparisons of multiple groups were done using one-way analysis of variation (ANOVA) and Kruskall Wallis tests for parametric and non-parametric variables, respectively. Spearman's and Pearson's correlation tests were used as appropriate for correlating non-parametric and parametric variables. For all tests, a probability (*P*) < 0.05 was considered significant.

## Results

Table 1 showed the demographic characteristics of the patients and controls. There were no significant differences regarding gender, age, weight, height and BMI between patients and controls (*P *> 0.05 for all). No significant difference was found between patient's subgroups regarding disease onset, 1^st ^blood transfusion onset, disease duration, blood transfusion duration, splenectomy duration, duration between splenectomy and 1^st ^blood transfusion, puberty age and family history of thalassemia (*P *> 0.05 for all) (Table 2).

**Table 1:** The demographic and anthropometric characteristics of blood transfusion-dependant β-thalassemia major patients and controls.

Parameters	Patients (n = 69)	Controls (n = 15)	*P*-value <
**Gender **(Male/Female)	45 (65.20%)/24 (34.80%)	11(73.30%)/4 (26.70%)	0.390

**Age **(years)	8.72 ± 3.70 (1.00-16.00)	8.40 ± 4.10 (3.00-14.00)	0.799

**Weight **(kg)	22.84 ± 7.04 (8.00-47.00)	23.30 ± 9.38 (11.00-40.00)	0.830

**Height **(meter)	1.16 ± 0.15 (0.80-1.47)	1.15 ± 0.18 (0.90-1.35)	0.941

**Body mass index **(kg/m2)	16.59 ± 2.13 (12.5-22.22)	16.80 ± 3.38 (12.19-22.40)	0.759

Data presented are mean +/- SD (range) or number (%); *P*-value, significance versus controls.

**Table 2:** The clinical characteristics of blood transfusion-dependant-thalassemia major patients.

Parameters	All patients	Patients subgroups		*P*-value <
			
	(n = 69)	With chelation [Group I] (n = 34)	Without chelation [Group II] (n = 35)	
**Disease onset age **(months)	13.74 ± -14.55 (2.00-60.00)	12.59 ± 12.77 (2.00-60.00)	14.86 ± 16.21 (2.00-60.00)	0.202

**Disease duration **(months)	87.42 ± 42.95 (3.00-180.00)	103.21 ± 38.95 (26.00-180.00)	72.09 ± 41.34 (3.00-174.00)	0.991

**1^st ^blood transfusion onset **(months)	13.74 ± 14.55 (2.00-60.00)	12.59 ± 12.77 (2.00-60.00)	14.86 ± 16.21 (2.00-60.00)	0.202

**Blood transfusions/year**	9.68 ± 2.96 (6.00-15.00)	9.65 ± 2.79 (6.00-12.00)	9.71 ± 3.16 (6.00-15.00)	0.202

**Splenectomy**	40 (58.00%)	23 (67.60%)	17 (48.90%)	0.464

**Splenectomy duration **(months)	37.75 ± 22.43 (3.00-96.00)	42.87 ± 20.76 (12.00-96.00)	30.82 ± 23.35 (3.00-84.00)	0.087

**Duration between splenectomy & 1^st ^blood transfusion **(months)	62.10 ± 35.28 (6.00-150.00)	69.48 ± 33.16 (6.00-137.00)	52.12 ± 36.57 (6.00-150.00)	0.520

**Puberty age **(years)	12.28 ± 0.46 (12.00-13.00)	12.17 ± 0.39 (12.00-13.00)	12.50 ± 0.55 (12.00-13.00)	0.055

**Positive family history**	13 (18.80%)	9 (26.50%)	4 (11.40%)	0.098

Data presented are mean +/- SD (range) or number (%); *P*-value, significance between patients with and without chelation therapy.

In patients with and without chelation therapy, significant higher levels of serum levels of CysC (*P *< 0.0001, *P *< 0.022), creatinine (*P *< 0.001, *P *< 0.008), uric acid (*P *< 0.0001, *P *< 0.002), inorganic phosphorus (*P *< 0.001, *P *< 0.001), while significant lower levels of TAC (*P *< 0.0001, *P *< 0.001), eGFR_Schwartz _(*P *< 0.001, *P *< 0.001) were reported than controls. In patients with chelation therapy, level of S_CysC _(*P *< 0.001) was higher while, S_TAC _was lower (*P *< 0.048) than patients without chelation therapy. Impaired renal functions (eGFR < 90 mL/minute/1.73 m^2^) was reported in 58.82% and 45.71% of patients with and without chelation (Table 3).

**Table 3:** Biochemical parameters in serum of β-thalassemia major patients' groups and controls.

Parameters	Controls	Patient groups	
		
	(n = 15)	With chelation [Group I] (n = 34)	Without chelation [Group II] (n = 35)
**Cystatin-C **(mg/L)	0.83 ± 0.05	1.26 ± 0.34	1.03 ± 0.26
Range	(0.80 - 0.90)	(0.85-2.00)	(0.82-2.12)
Significance		***P *< 0.0001**	*P *< 0.022, *P < 0.001

**Creatinine **(mg/dL)	0.55 ± 0.08	0.75 ± 0.18	0.70 ± 0.21
Range	(0.50 -0.70)	(0.50-1.10)	(0.40-1.10)
Significance		***P *< 0.001**	***P *< 0.008**, *P < 0.262

**eGFR_Schwartz _**(mL/min/1.73 m^2^)	119.08 ± 11.13	92.67 ± 25.16	93.73 ± 25.53
Range	(96.25-125.40)	(53.17-125.40)	(49.50-125.40)
Significance		***P *< 0.001**	***P *< 0.001**, *P < 0.852

≥90 mL/minute/1.73 m^2^	15 (100.00%)	14 (41.18%)	19 (54.29%)
< 90 mL/minute/1.73 m^2^		20 (58.82%)	16 (45.71%)

**Uric acid **(mg/dL)	3.81 ± 1.19	5.69 ± 1.69	5.30 ± 1.49
Range	(2.50-5.50)	(2.00-8.50)	(2.50-7.50)
Significance		***P *< 0.0001**	***P *< 0.002**, *P < 0.292

**Calcium **(mg/dL)	9.35 ± 1.11	9.10 ± 1.41	8.59 ± 1.86
Range	(8.01-10.95)	(6.09-11.10)	(5.35-12.37)
Significance		*P *< 0.121	*P *< 0.607, *P < 0.183

**Inorganic phosphate **(mg/dL)	3.32 ± 0.72	4.69 ± 0.87	4.50 ± 1.14
Range	(2.38-4.40)	(3.60-7.11)	(2.31-6.92)
Significance		***P *< 0.001**	***P *< 0.001**, *P < 0.412

**Total antioxidant capacity **(mmol/L)	0.43 ± 0.12	0.18 ± 0.10	0.23 ± 0.13
Range	(0.24-0.60)	(0.03-0.36)	(0.02-0.41)
Significance		***P *< 0.0001**	*P *< 0.0001, *P < 0.048

Data presented are mean +/- SD (range) or number (%); eGFR, estimated glomerular filtration rate. *P*-value, significance versus controls; *P-value, significance versus patients with chelation therapy.

In patient with and without chelation therapy, elevated urinary levels of NAG/Cr (*P *< 0.000, *P *< 0.002), β_2_MG/Cr (*P *< 0.005, *P *< 0.010), MDA/Cr (*P *< 0.021, *P *< 0.045) and albumin/Cr (*P *< 0.003, *P *< 0.006), uric acid (*P *< 0.008, *P *< 0.023), calcium (*P *< 0.003, *P *< 0.011), inorganic phosphorus (*P *< 0.001, *P *< 0.006) were reported than those of controls. Meanwhile, no significant difference was found in the levels of the urinary measured parameters between patients with and without chelation therapy. In patients with and without chelation therapy, normal urinary albumin (< 2.5 mg/mmol.Cr) was found in (5.90% and 28.60%%, respectively); microalbuminurea (2.5-30 mg/mmol.Cr) in (47.10% and 25.70%, respectively) and proteinurea (>30 mg/mmol.Cr) in (47.10% and 45.70%%, respectively) (Table 4).

**Table 4:** Biochemical parameters in urine of β-thalassemia major patients' groups and controls.

Parameters	Controls	Patients groups	
		
	(n = 15)	With chelation [Group I] (n = 34)	Without chelation [Group II] (n = 35)
**Albumin/Cr **(mg/mmol)	1.56 ± 0.51	68.19 ± 79.85	63.35 ± 79.89
Range	(0.50-2.00)	(1.20-216.00)	(0.50-300.00)
Significance		***P *< 0.003**	***P *< 0.006**, *P < 0.779

Normal albumin, < 2.5 mg/mmol.creatinine	15 (100.00%)	2 (5.90%)	10 (28.60%)
Microalbuminurea,2.5-30 mg/mmol.creatinine	-	16 (47.10%)	9 (25.70%)
Proteinuria, > 30 mg/mmol.creatinine	-	16 (47.10%)	16 (45.70%)

**NAG/Cr **(U/mg)	7.47 ± 2.28	15.00 ± 6.17	13.84 ± 7.60
Range	(3.00-9.50)	(6.00-25.00)	(3.00-30.00)
Significance		***P *< 0.000**	***P *< 0.002**, *P < 0.453

**β_2_MG/Cr **(ug/mg)	11.40 ± 6.70	70.90 ± 71.40	65.40 ± 74.00
Range	(1.80-22.00)	(3.00-220.00)	(0.90-280.00)
Significance		***P *< 0.005**	***P *< 0.010**, *P < 0.730

**Uric acid **(mg/dL)	25.20 ± 5.62	38.97 ± 18.53	36.86 ± 17.05
Range	(16.00-32.00)	(15.00-70.00)	(16.00-70.00)
Significance		***P *< 0.008**	***P *< 0.023**, *P < 0.593

**Calcium **(mg/dL)	9.01 ± 1.20	13.24 ± 4.53	12.63 ± 5.26
Range	(7.50-23.00)	(7.50-23.00)	(7.00-25.00)
Significance		***P *< 0.003**	***P *< 0.011**, *P < 0.569

**Inorganic phosphorus **(mg/dL)	11.83 ± 7.42	48.32 ± 38.83	42.53 ± 38.04
Range	(6.00-25.00)	(6.00-110.00)	(5.00-120.00)
Significance		***P *< 0.001**	***P *< 0.006**, *P < 0.495

**MDA/Cr **(nmol/mg)	13.57 ± 3.69	21.29 ± 9.77	20.20 ± 12.93
Range	(2.50-18.00)	(10.00-41.00)	(4.00-50.00)
Significance		***P *< 0.021**	***P *< 0.045**, *P < 0.671

Data presented are mean +/- SD (range) or number (%); Cr, creatinine; NAG, N-acetyl-beta-D-glucosaminidase; β_2_MG, β_2_-microglobulin; MDA, malondialdehyde; *P*-value, significance versus controls; *P-value, significance versus patients with chelation therapy.

In thalassemia patients, S_Cr _showed significant positive correlation with S_UA_, S_PO4 _(r = 0.391, *P *< 0.010; r = 0.339, *P *< 0.010) and negative correlation with S_Cr _(r = -0.309, *P *< 0.010). S_UA _showed significant positive correlation with U_β2MG_/Cr (r = 0.349, *P *< 0.010). eGFR_Schwartz _showed significant positive correlation with age, height, weight and S_TAC _(r = 0.374, *P *< 0.002; r = 0.319, *P *< 0.008; r = 0.300, *P *< 0.012; r = 0.313, *P *< 0.009) and negative correlation with S_PO4_, S_UA_, S_CysC _(r = -0.298, *P *< 0.013; r = -0.349, *P *< 0.003; r = -0.517, *P *< 0.001). S_CysC _showed significant positive correlation with S_Cr _(r = 0.659, *P *< 0.001) and negative correlation with S_TAC _(r = -0.295, *P *< 0.014). U_albumin_/Cr showed significant positive correlation with S_UA_, U_NAG_/Cr, U_MDA_/Cr, U_β2MG_/Cr (r = 0.339, *P *< 0.010; r = 0.885, *P *< 0.001; r = 0.845, *P *< 0.001; r = 0.991, *P *< 0.001). U_NAG_/Cr showed significant positive correlation with U_MDA_/Cr, U_β2MG_/Cr (r = 0.845, *P *< 0.001; r = 0.900, *P *< 0.001). U_MDA_/Cr showed significant positive correlation with U_β2MG_/Cr and S_UA _(r = 0.861, *P *< 0.001; r = 0.251, *P *< 0.050) (Table 5).

**Table 5:** Correlation coefficient (r) among various parameters in β-thalassaemia major (βTM) pediatric patients.

Parameters	Duration of disease	Age	BMI	S_CysC_	S_Cr_	S_TAC_	S_UA_	S_Ca_	S_PO4_	eGFR	U_NAG/Cr_	U_B2MG/Cr_	U_MDA/Cr_
**Age**	0.941***												

**BMI**	0.387**	0.356**											

**S_CysC_**	0.168	0.101	0.046										

**S_Cr_**	0.051	-0.078	‐0.027	0.659***									

**S_TAC_**	0.161	0.042	0.193	-0.293*	-0.309**								

**S_UA_**	0.073	0.002	-0.091	0.142	0.319**	-0.077							

**S_Ca_**	-0.101	-0.077	-0.047	0.091	-0.169	0.105	0.078						

**S_PO4_**	0.054	0.023	0.055	0.150	0.339**	0.212	0.055	-0.325**					

**eGFR**	0.229	0.374**	0.097	-0.517**	-0.892***	0.312**	-0.349**	0.144	-0.298*				

**U_NAG/Cr_**	0.070	0.144	-0.021	0.141	0.159	0.014	0.220	0.020	0.020	-0.083			

**U_B2MG/Cr_**	0.036	0.117	-0.081	0.094	0.0161	-0.018	0.349**	-0.076	0.076	-0.096	0.900***		

**U_MDA/Cr_**	0.010	0.068	-0.072	0.142	± 0.188	0.041	0.251*	0.023	0.108	-0.091	0.853***	0.861***	

**U_albumin/Cr_**	0.089	0.088	-0.105	0.071	0.146	0.024	0.339**	-0.084	0.085	-0.088	0.885***	0.991***	0.845***

Significance: *: *P < 0.05*; **: *P *< 0.01; ***: *P *< 0.001

BMI, body mass index; S_CysC_, serum cystatin-C; S_Cr_, serum creatinine, S_TAC_, serum total antioxidant capacity; S_UA_, serum uric acid; S_Ca_, serum calcium; S_PO4_, serum inorganic phosphorus; eGFR, estimated glomerular filtration rate; U_NAG/Cr_, urinary N-acetyl-beta-D-glucosaminidase to creatinine; U_B2MG/Cr_, urinary β_2_-microglobulin to creatinine; U_MDA/Cr_, urinary malondialdehyde to creatinine; U_albumin/Cr_, urinary albumin to creatinine.

## Discussion

Several investigations about renal involvement in adult βTM patients [4,5,7,16,17] were reported but there is a dearth of pediatric data. This study aimed to investigate the presence of glomerular and/or tubular dysfunctions in children and adolescent with TD- βTM, without any overt renal diseases, using both conventional and early markers of glomerular and tubular dysfunctions, and to correlate the findings to oxidative stress markers and iron chelation therapy with DFO. Results of this study showed impaired in classical (Cr, albumin and eGFR) and early (CysC) glomerular function markers in βTM patients than controls. Mean serum level of Cr was significantly higher while eGFR was significantly lower in patients with and without chelation therapy than controls. Impaired eGFR (< 90 mL/minute/1.73 m^2^) was found in 58.82% and 45.71% of patients with and without chelation therapy. In this respect, Grundy et al. [18] reported high serum level of Cr in βTM patients. In contrast, Li Volti et al. [19] and Aldudak et al. [5] found normal S_Cr_, and creatinine clearance (CrC) in patients who received subcutaneous DFO treatment. In consistence with others [20], we did not find any variation of serum Cr with age, meanwhile, Helin et al. [21] found a positive correlation between age and Cr. Stevens and Levey [11] found a positive correlation between eGFR and age, height, weight and 1^st ^blood transfusion age in thalassemia patients. In accordance, microalbuminurea and proteinurea were reported in our patients with (47.10% and 47.10%) and without chelation therapy (25.70% and 45.70%). Albuminurea found in this study could be attributed mainly to destruction of glomerular filtration membrane which proven by decreased in eGFR as well as tubulopathy. In accordance, Mohkam et al. [22] found proteinuria in 89.3% of βTM patients. Katopodis et al. [23] reported proteinuria and microalbuminuria in patients with sickle cell beta-thalassemia and related these finding to prolonged glomerular hyperfiltration, glomerulosclerosis, prostaglandin secretion and chronic anemia.

Cystatin-C is a 122-amino acid non-glycosylated low molecular weight (13 kDa) protein that inhibits cysteine proteases. It is considered to be a housekeeping gene since it is transcribed at a relatively constant level and is expressed in all nucleated cells. CysC is filtered by glomeruli and is followed by tubular reabsorption and degradation resulting in excretion of a minute amount in the urine. It is not secreted by the renal tubules and not reabsorbed back into the serum. Therefore, its serum levels serve as an endogenous parameter of GFR [24-26]. In this study, S_CysC _was significantly higher in patients with and without chelation therapy than controls. Also, S_CysC _showed significant positive correlation with S_Cr _but negative correlation with eGFR. In consistence, Voskaridou et al. [27] reported high S_CysC _levels in 32.1% of patients with sickle cell/beta-thalassemia. Economou et al. [28] reported 36% of their βTM patients had elevated S_CysC_. The data compared correlation coefficients between GFR and reciprocals of serum creatinine and CysC in 3,703 subjects, finding significantly better correlations for CysC and suggesting that CysC is superior to serum Cr for detection of impaired GFR in cross-sectional studies [24]. In consistence with others [20], we did not find any correlation of CysC with age, weight, height or gender. In contrast, MacDonald et al. [29] reported that S_CysC _is not independent of body composition, as previously thought. These authors showed that past studies were based on the fact that CysC is produced at a constant rate by all nucleated cells, but 60% of cellular mass comes from muscles, which varies with anthropometric measures, diet, genetics and physical activity.

The results of the present study also showed signs of renal tubulopathy, such as increase in serum levels uric acid and inorganic phosphorus and urinary excretion of albumin, uric acid, calcium and inorganic phosphorus in thalassaemic patients with and without chelation therapy compared to healthy controls. The increased serum and excretory levels of UA and PO_4 _can be explained by rapid erythrocyte turnover in combination with decreased reabsorption of filtered UA and PO_4 _from damaged renal tubules. Similarly, Lapatsanis et al. [30] reported phosphaturia and increase in serum phosphorus levels in βTM patients. In Turkey, Aldudak et al. [5], reported elevated urinary levels of protein/Cr and serum levels of UA and PO_4 _in βTM patients. Mohkam et al. [22] and Economou et al. [28] reported hypercalciuria in a large number of thalassemia patients. Smolkin et al. [31] found hyperuricosuria without hyperuricemia and hypercalciuria in βTM patients without relation to DFO treatment. The authors attributed hypercalciuria to hypoparathyroidism and related treatment with calcium and vitamin D [31]. Grundy et al. [18] reported lower serum levels of calcium and phosphate in children with βTM and contributed their findings to either liver or parathyroid glands involvement. In our studied patients, these findings reflect proximal tubular damage, as none of our patients suffered from liver diseases or hypoparathyroidism or received relative supplements.

N-acetyl-beta-D-glucosaminidase (EC 3.2.1.30) is a high molecular weight (140.000 Dalton) lysozomal enzyme. It is not specific to the kidney and found in other tissues as well. It cannot pass into glomerular ultrafilterate due to its high molecular weight. This enzyme shows high activity in renal proximal tubular cells. As, urinary NAG is not of plasmatic origin and is not filtered through the glomeruli, so enhanced U_NAG _is due to nonspecific release of tubular proteins as results of tubular dysfunction and tubular proteinuria and not secondary to loss of glomerular selectivity [27,32]. β_2_MG is a low molecular weight protein (11.8 kDa) which, under normal circumstances, is freely filtered at the glomerulus but almost totally reabsorbed and degraded by renal tubules [33]. Elevation of urinary β_2_MG is a sensitive and reliable early marker of tubular dysfunction [34,35]. In our study, the significant elevated excretion of NAG/Cr and β_2_MG/Cr in βTM patients with and without chelation therapy than those of controls indicate proximal tubular renal damage. In a similar way, others reported high U_NAG _activities [3,4,16,22,27,31] and β_2_MG excretion [27,28,36] in thalassemic patients. In this study, a significant positive correlation was found between U_NAG_/Cr and U_β2MG_/Cr ratios and between them and U_albumin_/Cr. In consistence, Mohkam et al. [22] found that proteinuria was positively correlated with U_NAG_/Cr ratio. As such, U_NAG _can be considered a sensitive and reliable index of proximal tubular dysfunctions in βTM patients.

The underlying mechanism for renal dysfunctions in patients with βTM is not clear. They seem to be multifactorial, attributed mainly to include long-standing anemia, chronic hypoxia, iron overload and deferoxamine toxicity [5,16,37]. In βTM patients with and without chelation, we found significant decreased serum levels of TAC (irrespective of high serum uric acid in our patients) and increase urinary excretion of MDA. Also, we found in thalassemia patients, S_TAC _was significantly positively correlated with eGFR, negatively with S_Cys_, S_Cr _and U_MDA_/Cr was positive significant correlation with each of U_NAG_/Cr, U_β2MG_/Cr and U_albumin_/Cr. This emphasis the role of oxidative stress in inducing renal tubular damage [5,38,39]. Although severe anemia and chronic hypoxia are believed to play a role in renal involvement in βTM, lipid peroxidation is currently the most favored hypothesis: according to this hypothesis, the imbalance in synthesis of hemoglobin (Hb) leads to excess unpaired globin chain and high intracellular content of non-Hb iron. The unstable Hb subunits are known to generate free oxygen radical species that starting a chain of oxidative events which leading to disintegration of denatured globin chains, heme, and iron, which bind to different membrane proteins and altering their normal structure and functions. In addition, the excess free iron is known to be a catalyst of lipid peroxidation via the fenton reaction [39].

Although transfusion therapy prolongs survival in βTM, the absence of a physiological iron excretion mechanism leads to uneven accumulation of this metal in various body organs results in death, usually during the second decade of life [40]. Therefore, lifelong chelation therapy, the most common of which is deferoxamine, which produces iron excretion in urine and stool, is an essential facet in the management of β-thalassemia patients [41]. The data obtained from this study showed decreased in both glomerular and tubular functions in βTM pediatric patients on a hypertransfusion protocol with regular DFO chelation therapy program. In addition, S_CysC _(indicator of glomerular dysfunction) was significantly higher while S_TAC _(indicator of total antioxidant capacity) was significantly lower in patients on DFO therapy than those without chelation therapy. Meanwhile, large number of patients on DFO therapy showed impaired eGFR (58.82%), microalbuminurea (47.10%) and proteinurea (47.10%) suggesting nephrotoxic effect of DFO. Although our patients received DFO subcutaneously in doses that are considered non-nephrotoxic according to a generally accepted protocol [42], the possibility that chronic administration of this agent caused duration dependent kidney damage cannot be excluded. In consistency, Koren et al. [6] reported that subcutaneous administration of DFO was associated with a clinically significant decrease in GFR in 40% of βTM patients and with a mild decrease in another 40%. Mohkam et al. [22] found a significant relationship between U_NAG _and duration of DFO therapy. On contrary, others [4,5,16-18,31] reported no detrimental effect of DFO on renal functions. On contrary, others [4] reported lower level of U_MDA _in groups treated with DFO than those without treatment which supports the hypothesis of direct suppressive effect of DFO on peroxidation.

## Conclusions

The presented data confirm that glomerular and tubular dysfunctions exist in children and adolescent with TD-βTM. These abnormalities are mainly sub-clinical, but, with prolonged repeated tubule damage, tubulointerstitial fibrosis may occur. As renal dysfunction may not be detected by routine tests, use of early markers is recommended. CysC is a promising marker of monitoring of glomerular dysfunction. Urinary NAG excretion can be considered a reliable index of the tubular toxicity and a possible predictor of proteinuria. In βTM, the renal dysfunction may be partially explained by excessive oxidative stress as well as by deferoxamine toxicity. Based on the results of our study, the usefulness of alternative chelation drugs and their effects on glomerular and tubular functions should be studied. The administration of selective antioxidants, along with an appropriate, nutritionally balanced diet would represent a promising approach towards counteracting oxidative damage and its deleterious effects on thalassemia.

## Competing interests

The authors declare that they have no competing interests.

## Authors' contributions

EAH designed the study, drafted the manuscript, carried out collection of serum and urine samples from patients, laboratory analysis of serum and urinary electrolytes and statistical analysis. NTM participated in the design of the study, drafted the manuscript and carried out the other laboratory analysis. Both authors read and approved the final manuscript.
